# Identification of the Prognostic Value of Tumor Microenvironment-Related Genes in Esophageal Squamous Cell Carcinoma

**DOI:** 10.3389/fmolb.2020.599475

**Published:** 2020-12-14

**Authors:** Donglei Zhang, Changlin Qian, Huabing Wei, Xiaozhe Qian

**Affiliations:** ^1^Department of Thoracic Surgery, Renji Hospital, School of Medicine, Shanghai Jiaotong University, Shanghai, China; ^2^Department of General Surgery, Renji Hospital, School of Medicine, Shanghai Jiaotong University, Shanghai, China

**Keywords:** esophageal squamous cell carcinoma, tumor microenvironment, prognosis, overall survival, TCGA

## Abstract

**Background:** Esophageal squamous cell carcinoma (ESCC) is the most prevalent histological type of esophageal cancer, but there is a lack of definite prognostic markers for this cancer.

**Methods:** We used the ESTIMATE algorithm to access the tumor microenvironment (TME) of ESCC cases deposited in the TCGA database, and identified TME-related prognostic genes using Cox regression analysis. A least absolute shrinkage and selector operation or LASSO algorithm was used to identify key prognostic genes. Risk scores were calculated, and a clinical predictive model was constructed to evaluate the prognostic value of TME-related genes.

**Results:** We found that high immune and stromal scores were significantly associated with poor overall survival (*p* < 0.05). We identified a total of 1,151 TME-related differently expression genes, among which 67 were prognosis-related genes. Through the LASSO method, 13 key prognostic genes were selected, namely, *ADAMTS16, LOC51089, CH25H, CORO2B, DLGAP1, GYS2, HAL, MXRA8, NPTX1, OTX1, RET, SLC24A2*, and *SPI1*, and a 13-gene risk score was constructed. A higher score was indicative of a poorer prognosis than a lower risk score (hazard ratio = 8.21, 95% confidence interval: 2.56–26.31; *P* < 0.001). The risk score was significantly correlated with immune/stromal scores and various types of infiltrating immune cells, including CD8 cells, regulatory T cells, and resting macrophages.

**Conclusion:** We characterized the tumor microenvironment in ESCC, and identified the key prognosis genes. The risk score based on the expression profiles of these genes is proposed as an indicator of TME status and is instrumental in predicting patient prognosis.

## Introduction

Esophageal cancer is the seventh most common cause of cancer-related deaths worldwide (Fitzmaurice et al., 2018). Esophageal squamous cell carcinoma (ESCC) accounts for ~90% of all esophageal cancers, which are always in the advanced stage at the time of their first diagnosis (Rustgi and El-Serag, 2014). Although recent advances in therapeutic approaches for gastroesophageal tumors have significantly improved the curative resection rates, as well as the disease-free and overall survival rates, the prognosis of patients with ESCC remains unfavorable (Cunningham et al., 2006; Baba et al., 2018). The pathological subtypes of this cancer present limitations in the prediction of prognosis, as patients with similar clinical and pathological types could have totally different final outcomes (Smyth et al., 2017). Thus, it is crucial to identify prognostic biomarkers for ESCC patients and develop more effective therapies.

The tumor microenvironment (TME) is composed of stromal cells, immune cells, extracellular matrix molecules, and inflammatory mediators. Tumor cells could promote immune escape by forming an immunosuppressive microenvironment (Hegde and Chen, 2020). In addition to the genetic heterogeneity of tumor cells, the heterogeneity of immune and stromal cells also contribute to the complexity of TME; this affects the time and intensity of the anti-tumor response and becomes a major obstacle in the treatment of tumors. Even though much attention has been devoted to the role of TME in the development of cancers and their clinical outcomes (Galon et al., 2012; Pitt et al., 2016; Senbabaoglu et al., 2016), little is known about the relationship between TME and the prognosis of ESCC patients, or the role of TME-related genes in ESCC.

In the present study, we gathered information about the clinical features and RNA sequencing data of 95 ESCC tumor samples from the TCGA database and evaluated their TME profiles. We then identified the correlation between the TME profile and patient prognosis. The relevant mechanism was explored with gene expression profiling, and a TME-related gene signature model was established for predicting the prognosis of ESCC.

## Materials and Methods

### Data Collection From the TCGA Database

Gene expression and clinical data of 185 esophageal cancer patients were downloaded from the TCGA database (https://portal.gdc.cancer.gov/). Only patients with a histological diagnosis of ESCC and who had not undergone neoadjuvant chemotherapy were included. Based on these criteria, 95 ESCC cases with gene expression data were included in our analysis. The clinical information of the patients is shown in Table 1.

**Table 1:** Clinical features of patients with esophageal squamous cell carcinoma in TCGA.

**Clinical features**	**Count (%) (*n* = 95)**
**Status**	
Alive	63 (66.3)
Dead	32 (33.7)
**Gender**	
Female	14 (14.7)
Male	81 (85.3)
**Age**	
≤50	21 (22.1)
>50	74 (77.9)
**Grade**	
G1	16 (16.8)
G2	48 (50.5)
G3	21 (22.1)
Missing	10 (10.5)
**Stage**	
Stage 1	7 (7.4)
Stage 2–3	82 (86.3)
Stage 4	4 (4.2)
Missing	2 (2.1)
**Stage T**	
T1	8 (8.4)
T2	31 (32.6)
T3	50 (52.6)
T4	4 (4.2)
Missing	2 (2.1)
**Stage** ***N***	
N0	54 (56.8)
N1	29 (30.5)
N2	6 (6.3)
N3	3 (3.2)
NX	1 (1.1)
Missing	2 (2.1)
**Stage M**	
M0	83 (87.4)
M1	4 (4.3)
MX	5 (5.3)
Missing	3 (3.2)

### Calculation of Stromal and Immune Score

The ESTIMATE algorithm was used to calculate immune and stromal scores for each tumor sample with the *estimate* R package (Yoshihara et al., 2013). The stromal, immune, and ESTIMATE scores were compared across different clinical indexes with the Wilcoxon rank-sum test (two groups) or Kruskal-Wallis *H*-test (three or more groups).

To determine the optimal cutoff value for grouping patients, maximally selected rank statistics were employed with the *maxstat* R package (Hothorn and Zeileis, 2008). Based on the cutoff value, all samples were divided into the high and low score groups.

### Identification of Differentially Expressed Genes and Functional Enrichment Analysis

Linear models were used to identify differentially expresses genes (DEGs) between the two immune/stromal groups (high score group vs. low score group) using the *limma* R package (Ritchie et al., 2015). A false discovery rate (FDR) adjusted *p* < 0.05 combined with a simultaneous absolute value of >1 for logFC was set as the threshold for DEG identification. Next, upregulated and downregulated genes were identified based on the stromal and immune scores.

Functional enrichment analysis of the DEGs with the Kyoto Encyclopedia of Genes and Genomes (KEGG) and gene ontology (GO) was carried out using the *clusterProfiler* R package (Yu et al., 2012). The GO terms are classified under biological process (BP), cellular component (CC), and molecular function (MF).

### Identification of Key Prognostic Genes

In order to identify key prognostic genes, we conducted a comprehensive analysis. First, we used a Cox regression analysis to estimate the association between the expression of all DEGs and the overall survival time of patients. Tumor samples were grouped into the “high expression group” and “low expression group” with the median gene expression level set as the cutoff. Through this method, 67 genes for which the *p* < 0.05 were identified as candidate prognostic genes.

Next, a least absolute shrinkage and selector operation (LASSO) algorithm was used to identify key prognostic genes with the *glmnet* R package (Friedman et al., 2010). Clinical variables, including age, sex, tumor grade, and tumor stage, were included. Lambda.min is the cutoff point at which the minimum mean cross-validated error occurs. Genes or indexes whose coefficient was not 0 at lambda.min were selected for further analysis.

### External Validation of Key Prognostic Genes

The genes selected by the LASSO method were further validated in a GEO dataset (GSE44021). We extracted 73 samples of ESCC tumor tissue from the dataset, and the immune and stromal scores were calculated based on their expression profile. The correlation of the prognostic genes selected by LASSO to the immune/stromal scores was evaluated utilizing a Spearman's correlation test.

### Risk Score Calculation and Model Construction

The risk scores for every tumor sample were calculated using the following formula:

$$\begin{array}{l} Risk\;score=\frac{\sum_{i=1}^{n}Exp_{i}\;*\;\frac{\pm 1}{D_{i}}}{\sum_{i=1}^{n}\frac{1}{D_{i}}} \end{array}$$

In the above formula, *Exp* represents the expression level of the gene, ± is the positive or negative sign for the regression coefficient of the gene calculated with the LASSO method, and *D* represents the variance in the expression level of the gene in all the samples.

To verify that this risk score can independently predict the prognosis of patients with ESCC, multivariate Cox survival analysis was performed with sex and age as covariates.

### Estimation of Immune Cell Fractions

CIBERSORT (https://cibersort.stanford.edu/) and leucocyte signature matrix 22 (LM22) were used to quantify the proportions of different immune cell types in the ESCC samples from the TCGA database (Newman et al., 2015). Normalized gene expression data were analyzed using the CIBERSORT algorithm by running 1,000 permutations. The CIBERSORT *p*-value reflects the statistical significance of the results, and a threshold <0.05 is recommended. Finally, samples with CIBERSORT *p* < 0.05 were included in the analysis of the correlation between risk scores and immune cell types.

### Statistical Analysis

The correlation between the risk scores and immune cell infiltration or TME scores were evaluated using Spearman's correlation analysis. Survival curves were compared using the Kaplan-Meier method and the log-rank test. A ROC curve was used to calculate the AUC value for 1 and 2-year survival. Hazard ratios (HRs) and 95% confidence intervals (CIs) were calculated using Cox regression analysis. All tests were two-sided, and a *p* < 0.05 was considered to indicate significance, unless stated otherwise. All analyses were performed with R version 4.0.2.

## Results

### Association of High Immune and Stromal Scores With Poor Prognosis

The stromal, immune, and ESTIMATE scores of all 95 ESCC tumor samples were calculated using the ESTIMATE algorithm based on their gene expression profile. The immune scores ranged from −1,190 to 2,705; the stromal scores, from −1,370 to 1,136; and the ESTIMATE scores, from −2,232 to 3,231. There was no significant difference in the immune scores according to any of the clinical indexes, and the stromal or ESTIMATE scores did not show significant differences either (Supplementary Figure 1).

**Figure 1:**
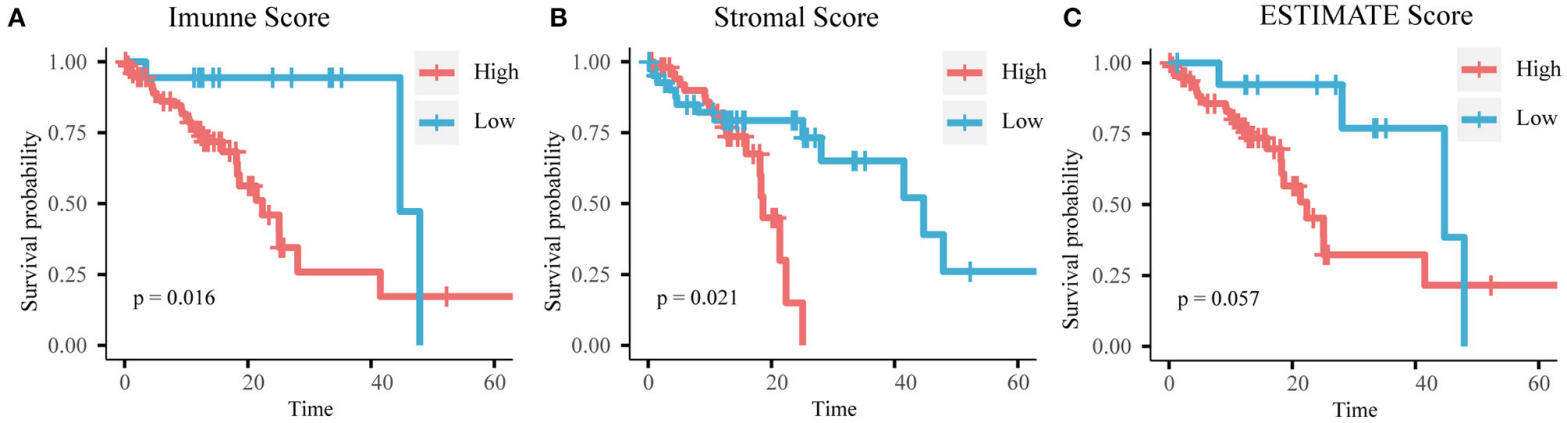
Kaplan-Meier curves of **(A)** immune score, **(B)** stromal score, and **(C)** ESTIMATE score with overall survival time.

To determine whether there was a correlation between the stromal/immune/ESTIMATE scores and overall survival, we separated samples into high- and low-score groups according to the cutoff values determined by maximally selected rank statistics (Supplementary Figure 2). According to the immune scores, 77 patients were assigned to the high score group and 22 patients were assigned to the low score group. According to the stromal scores, 53 patients were assigned to the high score group and 42 patients were assigned to the low score group. According to the ESTIMATE scores, 81 patients were assigned to the high score group and 14 patients were assigned to the low score group. Kaplan-Meier survival analysis showed that a high immune score and high stromal score were associated with poor overall survival (Figure 1).

### Identification of DEGs and Functional Analysis

**Figure 2:**
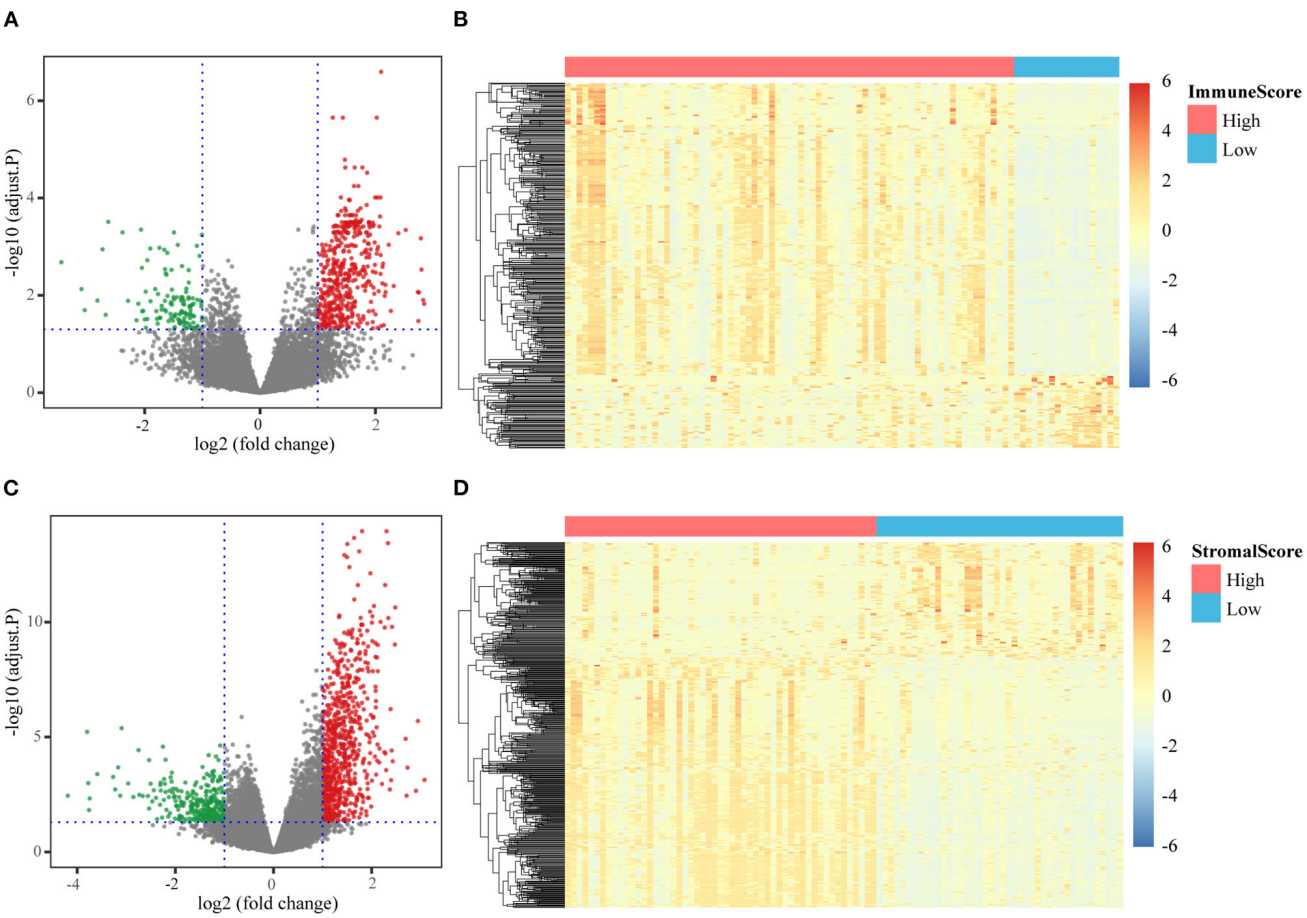
Different expression genes identification. **(A)** Volcanic diagram of DEGs based on the comparison of high/low immune scores; **(B)** expression levels of DEGs based on the comparison of high/low immune scores; **(C)** volcanic diagram of DEGs based on the comparison of high/low stromal scores; **(D)** expression levels of DEGs based on the comparison of high/low stromal scores.

Differential expression analysis was performed between the high- and low-score groups. When the threshold of |log2FC| and FDR were set at >1 and <0.05, respectively, 459 upregulated genes and 124 downregulated genes were identified based on the immune scores (Figure 2A), and 767 upregulated genes and 285 downregulated genes were identified based on the stromal scores (Figure 2B). Using hierarchical clustering analysis, we found that these DEGs could be used to distinguish tumors with high stromal/immune scores from those with low scores (Figures 2C,D).

All the DEGs were included in GO and KEGG functional enrichment analyses. The DEGs based on the immune score, were mainly enriched in BP related to immune response and immune cell activation, CC was associated with immune receptor activity, and MF terms were associated with the external side of the plasma membrane (Supplementary Figure 3A). The top KEGG pathway identified was the hematopoietic cell lineage pathway (Supplementary Figure 3C). The DEGs based on the stromal score were mainly enriched in BP terms that were strongly linked to extracellular matrix organization and extracellular structure organization, in CC terms that were associated with the collagen-containing extracellular matrix, and MF terms that were significantly related to extracellular matrix structural constituents (Supplementary Figure 3B). The top KEGG pathway identified was the cell adhesion molecule pathway (Supplementary Figure 3D).

### Identification of Key Prognostic Genes Based on the Machine Learning Method

To evaluate the prognostic effect of DEGs, Cox regression analysis was performed on the expression of 1,514 DEGs and overall survival. Finally, 67 genes were found to have a significant association with overall survival (Supplementary Table 1).

**Figure 3:**
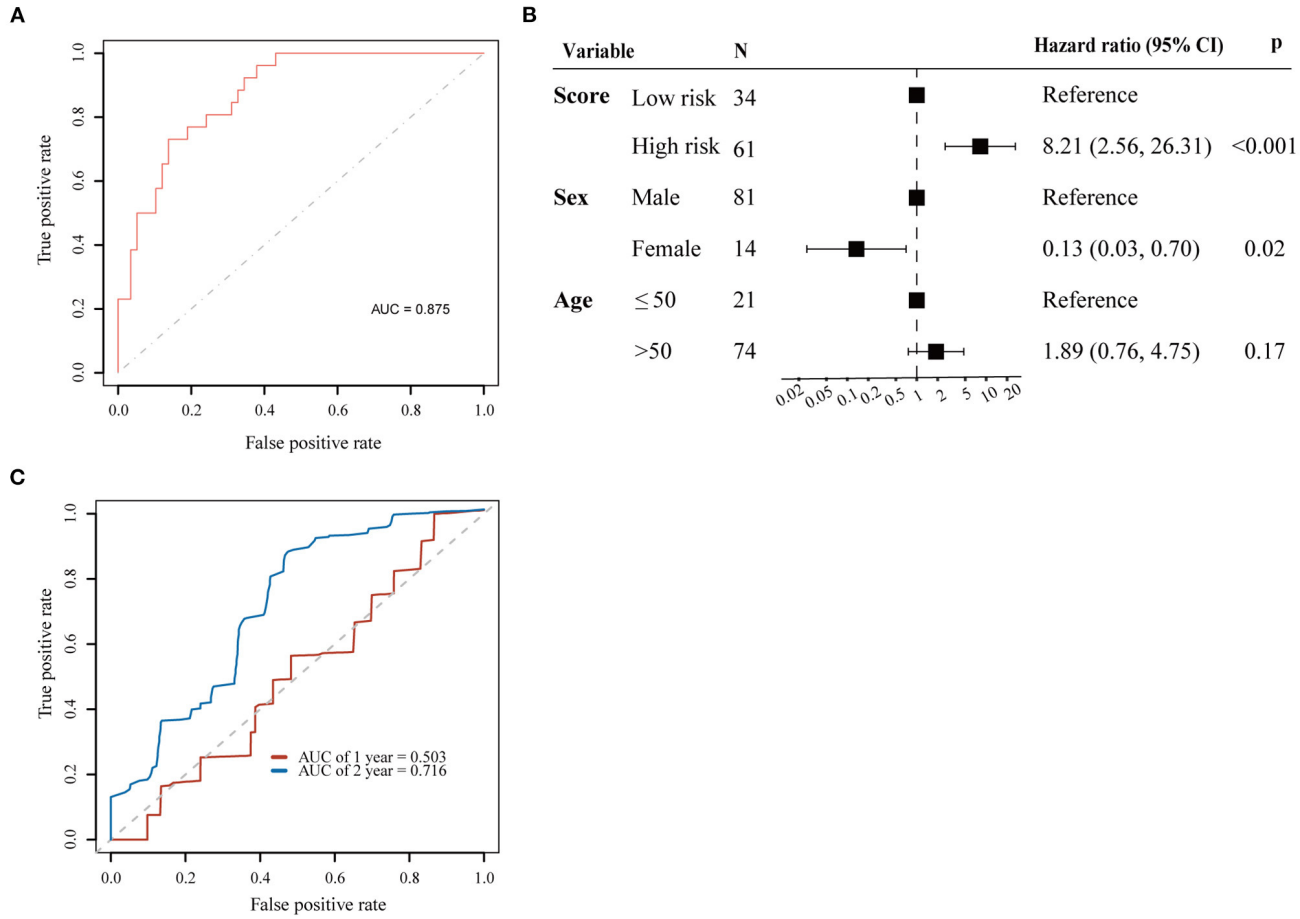
Prognostic value for 13 genes selected by LASSO. **(A)** ROC curve for 13 key prognostic genes to survival status; **(B)** Cox proportional hazards regression analyses results for the 13-gene score; **(C)** ROC curves for predicting 1 and 2-year overall survival probability with the 13-gene score.

We used the LASSO method to select key prognostic genes from these 67 candidate genes based on the clinical features (Supplementary Figure 4). Thirteen genes and the clinical indicators sex and age were identified (Table 2). Furthermore, the ability of these 15 features to predict survival was determined using the cv.glmnet function. ROC curve analysis revealed that the AUC value was 0.875 (Figure 3A).

**Table 2:** Thirteen key prognostic genes and two clinical indexes selected by the LASSO method.

**Clinical index/gene**	**Description**	**Coefficient**	**Hazard ratio**	**95% CI**	***P*-value**
Age		0.0408			
Sex		−0.3281			
RET	Ret proto-oncogene	0.3421	1.25	1.04–1.50	0.02
LOC51089 (C1QA)	Complement C1q A chain	0.1787	1.34	1.04–1.72	0.02
CORO2B	Coronin 2B	0.2032	1.42	1.13–1.78	0.003
CH25H	Cholesterol 25–hydroxylase	0.1026	1.20	1.01–1.43	0.04
SPI1	Spi−1 proto–oncogene	0.1117	1.41	1.04–1.92	0.03
HAL	Histidine ammonia-lyase	0.0783	0.97	0.81–1.17	0.76
NPTX1	Neuronal pentraxin 1	0.0077	1.13	0.95–1.33	0.17
DLGAP1	DLG associated protein 1	−0.0174	0.95	0.81–1.11	0.53
OTX1	Orthodenticle homeobox 1	−0.0409	0.82	0.66–1.03	0.09
SLC24A2	Solute carrier family 24 member 2	−0.0341	0.69	0.52–0.91	0.008
ADAMTS16	ADAM metallopeptidase with thrombospondin type 1 motif 16	−0.1505	0.84	0.70–1.00	0.053
GYS2	Glycogen synthase 2	−0.2956	0.88	0.72–1.07	0.20
MXRA8	Matrix remodeling associated 8	−0.4201	0.72	0.56–0.93	0.01

*Multivariate Cox survival analysis was performed for every gene when sex and age were used as covariates*.

We used the 13 genes (*ADAMTS16, LOC51089, CH25H, CORO2B, DLGAP1, GYS2, HAL, MXRA8, NPTX1, OTX1, RET, SLC24A2*, and *SPI1)* selected by the LASSO algorithm for further investigation. We also analyzed the association between the expression of the 13 genes and overall survival using Cox regression analysis. Age and sex were inputted into each regression model. The high expression levels of *LOC51089* (HR = 1.34, 95% CI = 1.04–1.72)*, CH25H* (HR = 1.20, 95% CI = 1.01–1.43)*, CORO2B* (HR = 1.42, 95% CI = 1.13–1.78)*, RET* (HR = 1.25, 95% CI = 1.04–1.50), and *SPI1* (HR = 1.41, 95% CI = 1.04–1.92) were positively correlated with overall survival, while high expression levels of *MXRA8* (HR = 0.72, 95% CI = 0.55–0.93) and *SLC24A2* (HR = 0.69, 95% CI = 0.52–0.91) were negatively correlated with overall survival (Table 2). The correlation between the 13 genes and the infiltration of immune cells was identified by Pearson's correlation analysis (Supplementary Table 2).

### External Validation of Key Prognostic Genes

An external ESCC dataset from the GEO database was used to validate the correlation between the expression of the 13 key prognostic genes and the immune/stromal scores. Immune and stromal scores were calculated based on the gene expression profile, and 10 out of 13 genes were matched in this ESCC dataset. Among the 10 genes, the correlation of five genes to the immune/stromal scores was validated, including CH25H, HAL, MXRA8, SLC24A2, and SPI1 (Supplementary Figure 5).

### Construction of a 13-Gene Risk Score

The risk score was calculated based on the expression level (EL) of all 13 key prognostic genes. Risk score = [(−0.22 × EL of *ADAMTS16*) + (0.49 × EL of *LOC51089*) + (0.26 × EL of *CH25H*) + (0.42 × EL of *CORO2B*) + (−0.21 × EL of *DLGAP1*) + (−0.20 × EL of *GYS2*) + (0.25 × EL of *HAL*) + (−0.60 × EL of *MXRA8*) + (0.32 × EL of *NPTX1*) + (−0.53 × EL of *OTX1*) + (0.33 × EL of *RET*) + (−0.50 × EL of *SLC24A2*) + (0.83 × EL of *SPI1*)]/5.17. All the EL values were logarithmically transformed.

Patients were then separated into high-risk and low-risk groups according to the cutoff point of 0.98 by maximally selected rank statistics. To evaluate the risk score model based on the LASSO algorithm, Cox regression analysis was performed for overall survival time with sex, age, and risk group as covariates. The results of multivariate Cox analysis demonstrated that the risk score could be regarded as an independent predictive factor for OS (HR = 8.21, 95% CI = 2.56–26.31; *p* < 0.001) (Figure 3B).

Next, we established a prognostic nomogram to predict 1 and 2-year OS in 95 ESCC patients. The AUC values for 1 and 2-year OS were 0.503 and 0.716, respectively (Figure 3C).

### Correlation of Risk Score to TME-Score and Immune Cell Infiltration

**Figure 4:**
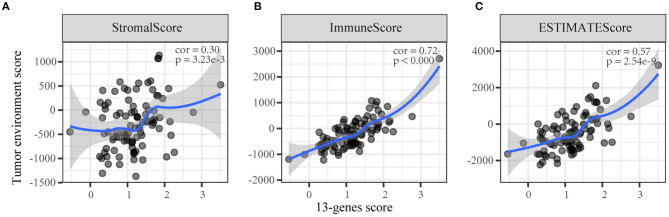
The correlation between the 13-gene score and tumor microenvironment scores. **(A)** Stromal score; **(B)** Immune score; **(C)** ESTIMATE score.

We conducted a Spearman's correlation test to evaluate the correlation between the TME score and the 13-gene risk score. The results showed that the 13-gene risk score was significantly correlated with the immune/stromal/ESTIMATE scores (Figure 4).

**Figure 5:**
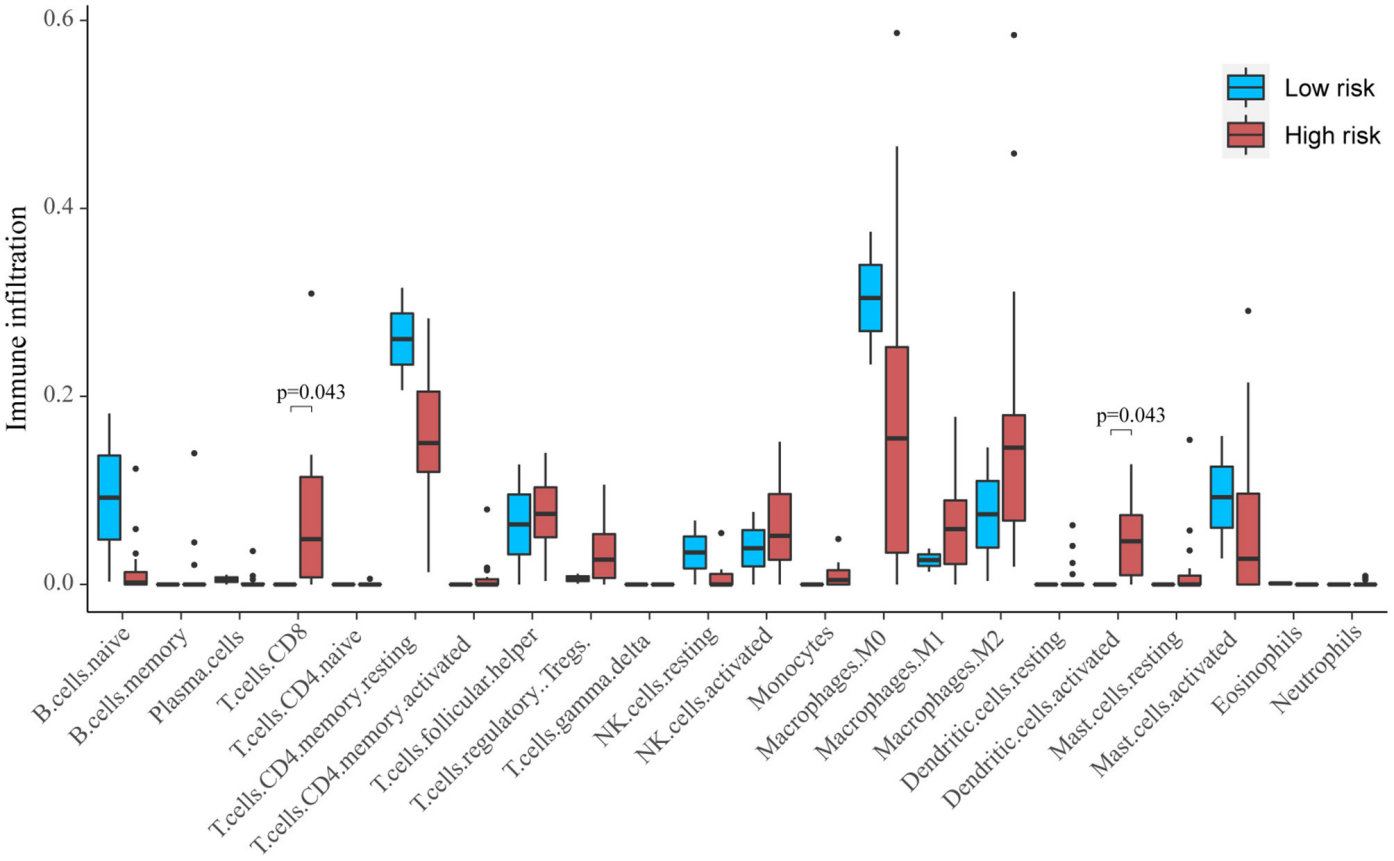
The profiles of immune infiltration between the high-risk and low-risk score groups.

**Figure 6:**
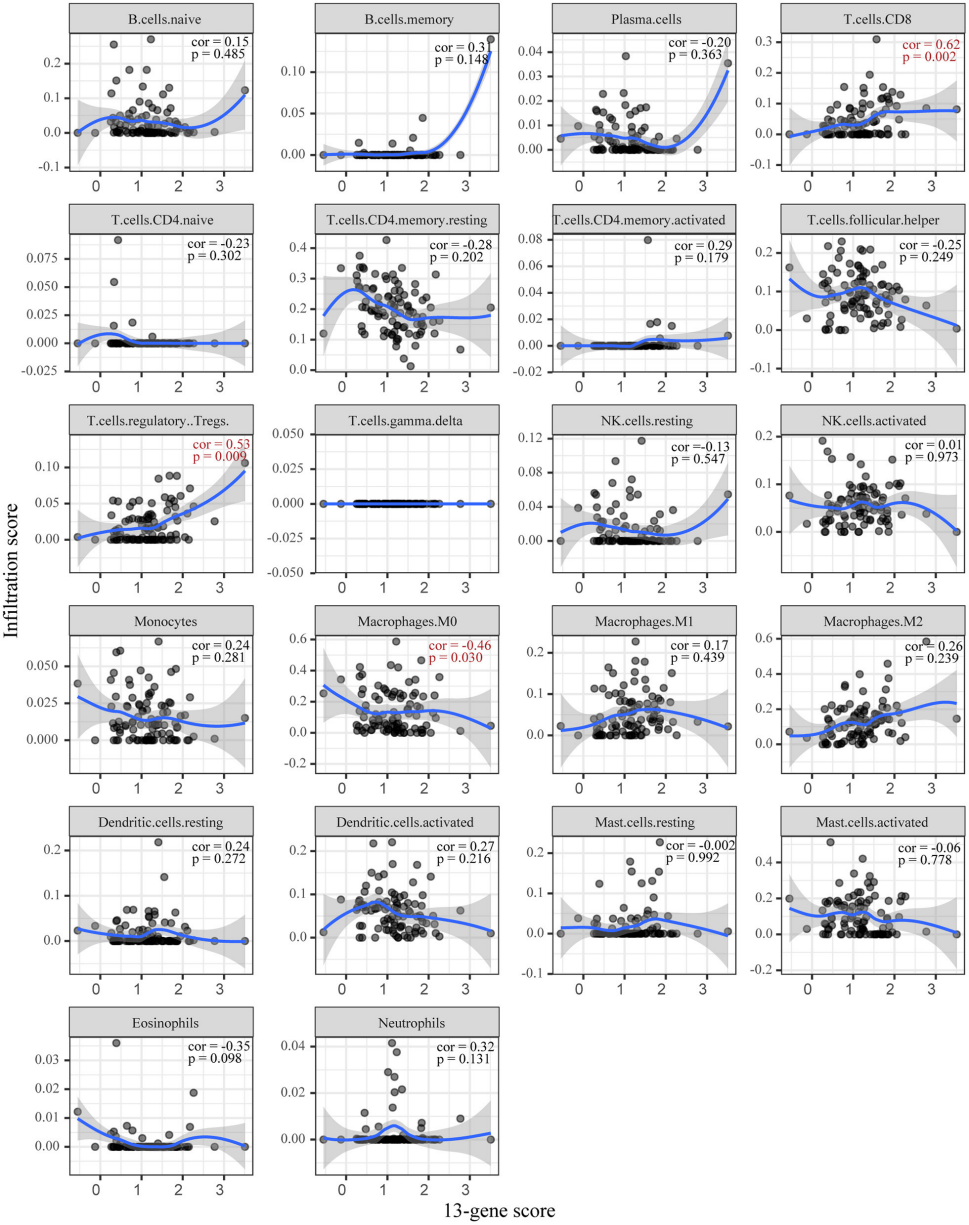
The correlation between the 13-gene score and immune infiltration.

Based on CIBERSORT, unmatched samples with *p* > 0.05 were removed. A total of 23 tumor samples, including 21 samples in the high-risk group and 2 samples in the low-risk group were used for further analysis. Our results showed that there were significant differences in the proportion of various immune cell fractions. The high-risk group had a higher infiltration of CD8 cells (*p* = 0.043) and dendritic cells (*p* = 0.043) (Figure 5). In addition, the correlation of the 13-gene risk score with CD8 cell, regulatory T cell, and resting macrophage (M0) fractions was confirmed using a Spearman's correlation test (Figure 6).

## Discussion

Although significant benefits of new therapeutic strategies have been reported in the past few decades, the overall survival rates of ESCC remain unsatisfactory (Cunningham et al., 2006; Baba et al., 2018). This is because ESCC is characterized by high heterogeneity that is attributable to the heterogeneity of tumor cells and the tumor environment, and as a result, the response to therapy is highly varied among patients (Marshall and Djamgoz, 2018). Therefore, it is critical to screen for prognostic markers related to the TME of ESCC. In the present study, we identified 13 TME-related genes and developed a risk score signature for ESCC. This signature could be used to efficiently determine the overall survival time of ESCC patients.

Previous studies have established several molecular models to predict the long-term survival of patients with ESCC (Wen et al., 2014; Chen W. et al., 2020; Wang C. et al., 2020). These studies often overlook the role of TME in ESCC. The ESTIMATE algorithm is a widely accepted and verified algorithm in studies on various cancers, such as endometrial cancer, cervical squamous cell carcinoma, and breast cancer (Priedigkeit et al., 2017; Pan et al., 2019; Chen P. et al., 2020). In this study, we calculated the stromal and immune scores of ESCC tumor tissues with the ESTIMATE algorithm in order to predict the level of the infiltration of stromal and immune cells. ESCC patients with high immune/stromal scores had a poorer prognosis than those with low scores; this indicates that the TME composition affects the clinical outcomes of ESCC patients. Furthermore, the DEGs based on immune score were significantly enriched in the regulation of T cell activation, the regulation of lymphocyte activation, and lymphocyte differentiation, while the DEGs based on stromal score were mainly enriched in extracellular matrix organization, extracellular structure organization, and cell-substrate adhesion. These findings confirm the role of immune cells and TME heterogeneity on the clinical outcomes of ESCC.

Cox regression analysis was performed to determine the association between the expression of DEGs and survival, and 67 DEGs were identified as being associated with overall survival. Then, using the LASSO algorithm, 13 key prognostic genes and two clinical indexes were selected. These genes had independent prognostic values, and the correlation of their expression to immune/stromal scores was also validated in the external GEO dataset. These genes have previously been reported to be associated with different malignant tumors in various ways. For example, abnormally methylated CH25H has been found to be a prognostic marker for lung squamous cell carcinoma patients (Gao et al., 2019). Additionally, low CH25H levels in leukocytes from melanoma patients were correlated with poor prognosis (Ortiz et al., 2019). CORO2B was found to be involved in many biological processes of malignant transformation in BEAS-2B cells induced by cigarette smoke (Wang et al., 2019). LOC51089, also called C1QA, was found to be involved in the innate immune system and was associated with the expression of PD-L1 (Olkhov-Mitsel et al., 2020), and its abnormal expression in tumor tissues was confirmed in head and neck squamous cell carcinoma and clear cell renal cell carcinoma (Yu et al., 2019; Apanovich et al., 2020). MXRA8 is one of the predicted tumor stroma-specific markers in various cancers (Kiflemariam et al., 2015). Accordingly, kidney renal clear cell carcinoma patients with high expressions of MXRA8 had worse overall survival (Li and Xu, 2019). RET is an important proto-oncogene that can undergo oncogenic activation through both cytogenetic rearrangement and the activation of point mutations, and alterations in RET have been identified as being oncogenic in multiple malignancies (Subbiah et al., 2020). Moreover, alterations in SLC24A2, a potassium-dependent sodium-calcium exchanger, were observed in pancreatic ductal adenocarcinoma (Wang et al., 2017). Alterations in SPI1 lead to cellular proliferation and differentiation arrest, resulting in oncogenic subversion (Roos-Weil et al., 2019). Finally, high expression levels of HAL contributed to poor prognoses in breast cancer patients (Wang C. Y. et al., 2020). Our study demonstrated that all these genes may be crucial biomarkers for predicting survival outcome in ESCC. Therefore, both *in vitro* and *in vivo* experiments are necessary to validate the expression of these genes and their roles in tumor cell proliferation, metastasis, and invasion. Further clinical studies are also required to determine whether these genes are independent prognosis biomarkers as well as their association with immunotherapy efficacy.

It is critical to accurately predict the prognosis of patients with ESCC for the selection of appropriate treatment. In this regard, combining different independent prognostic variables into one risk score can significantly improve prognostic potential. In this study, we constructed and verified a 13-gene risk score for ESCC based on stromal/immune scores. The 13-gene model can be used as a prognostic tool independently of other clinical and pathological features. Based on this signature, we could conveniently monitor the infiltration of immune cells and further reduce the degree of immune response. Thus, this signature could reflect these changes in TME from different aspects and has the potential to be appropriate for rational diagnosis and individualized treatment.

Several limitations of this study should be noted. First, given that the study was retrospective in nature, the risk scores need to be validated in a large cohort. Second, in this study, the associations between the 13 genes and the biological mechanisms of ESCC have not been clarified. Thus, further experiments are required to validate the exact mechanism of these 13 genes under *in vitro* and *in vivo* conditions.

## Conclusion

In conclusion, we developed a robust TME-related gene signature for the prognostic prediction of ESCC based on samples deposited in the TCGA database. Our signature could reflect the TME features and prognosis of ESCC. These findings are the basis for more studies on the specific roles of these TME-related genes in the development and progression of ESCC.

## Data Availability Statement

Publicly available datasets were analyzed in this study. This data can be found here: The Cancer Genome Atlas (https://portal.gdc.cancer.gov/) and Gene Expression Omnibus (GEO) Datasets (https://www.ncbi.nlm.nih.gov/).

## Author Contributions

DZ and CQ proposed the study concept, design, drafted the manuscript, collected, analyzed, and interpreted the data. XQ and HW participated in revising the manuscript. All authors contributed to the article and approved the submitted version.

## Conflict of Interest

The authors declare that the research was conducted in the absence of any commercial or financial relationships that could be construed as a potential conflict of interest.

## Abbreviations

ESCC: esophageal squamous cell carcinoma
TME: tumor microenvironment
LASSO: least absolute shrinkage and selector operation
HRs: hazard ratios
DEG: differently expression gene
CD8: cluster of differentiation 8
M0: resting macrophages.
