# Supplementary material for: Identification of the Prognostic Value of Tumor Microenvironment-Related Genes in Esophageal Squamous Cell Carcinoma
Source: Front Mol Biosci. 2020 Dec 14;7:599475. doi: 10.3389/fmolb.2020.599475 (PMC7767869; doi:10.3389/fmolb.2020.599475)
Supplement: Supplementary file 1 [file Data_Sheet_1.PDF]

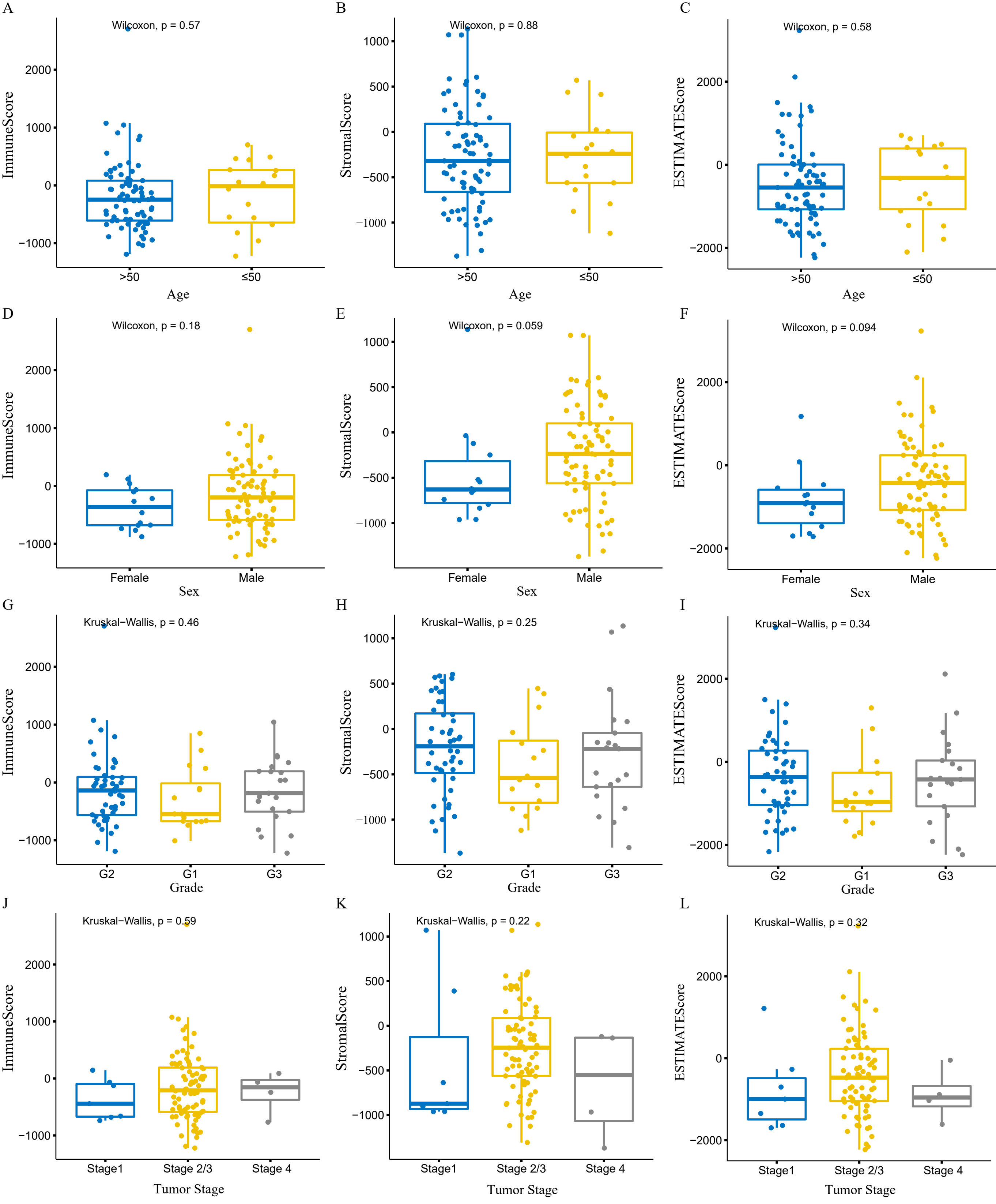

Supplementary Figure S1. Stromal scores, immune scores and ESTIMATE scores amoung age (A-C), sex (D-F), disease grade (G-I) and tumor stage(J-L) groups.
