# Supplementary material for: Identification of the Prognostic Value of Tumor Microenvironment-Related Genes in Esophageal Squamous Cell Carcinoma
Source: Front Mol Biosci. 2020 Dec 14;7:599475. doi: 10.3389/fmolb.2020.599475 (PMC7767869; doi:10.3389/fmolb.2020.599475)
Supplement: Supplementary file 2 [file Data_Sheet_2.PDF]

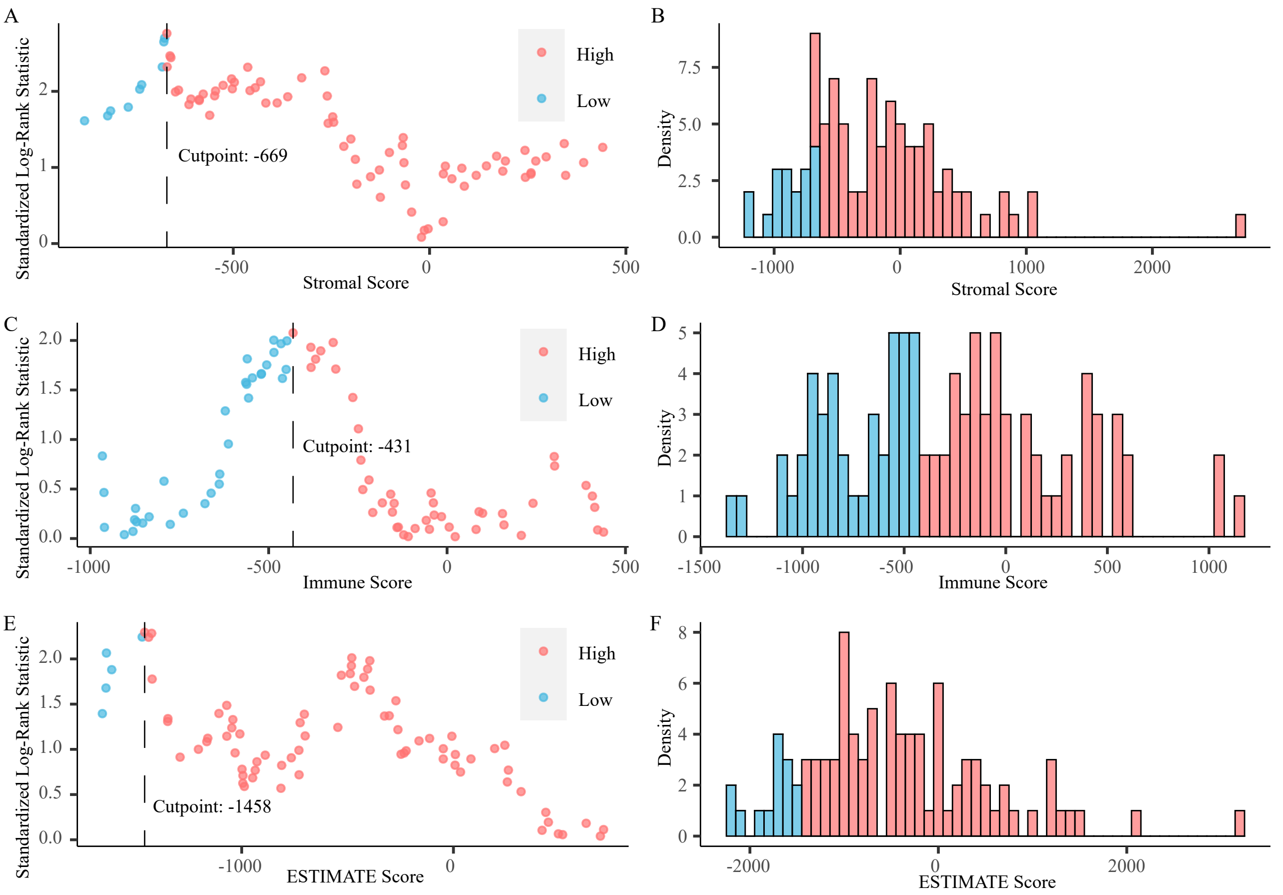

Supplementary Figure S2. The illustration of optimal cutoff identification for stromal score(A-B), immune score(C-D) and ESTIMATE score(E-F). The upper scatter plot shows the density distribution for low- and high-stromal/immune score groups divided by the optimal cutoff. The lower histogram shows the standardized log-rank statistic value for each corresponding expression cutoff. The optimal cutoff with the maximum standard log-rank statistic is marked with a vertical dashed line.
