# Supplementary material for: Identification of the Prognostic Value of Tumor Microenvironment-Related Genes in Esophageal Squamous Cell Carcinoma
Source: Front Mol Biosci. 2020 Dec 14;7:599475. doi: 10.3389/fmolb.2020.599475 (PMC7767869; doi:10.3389/fmolb.2020.599475)
Supplement: Supplementary file 3 [file Data_Sheet_3.PDF]

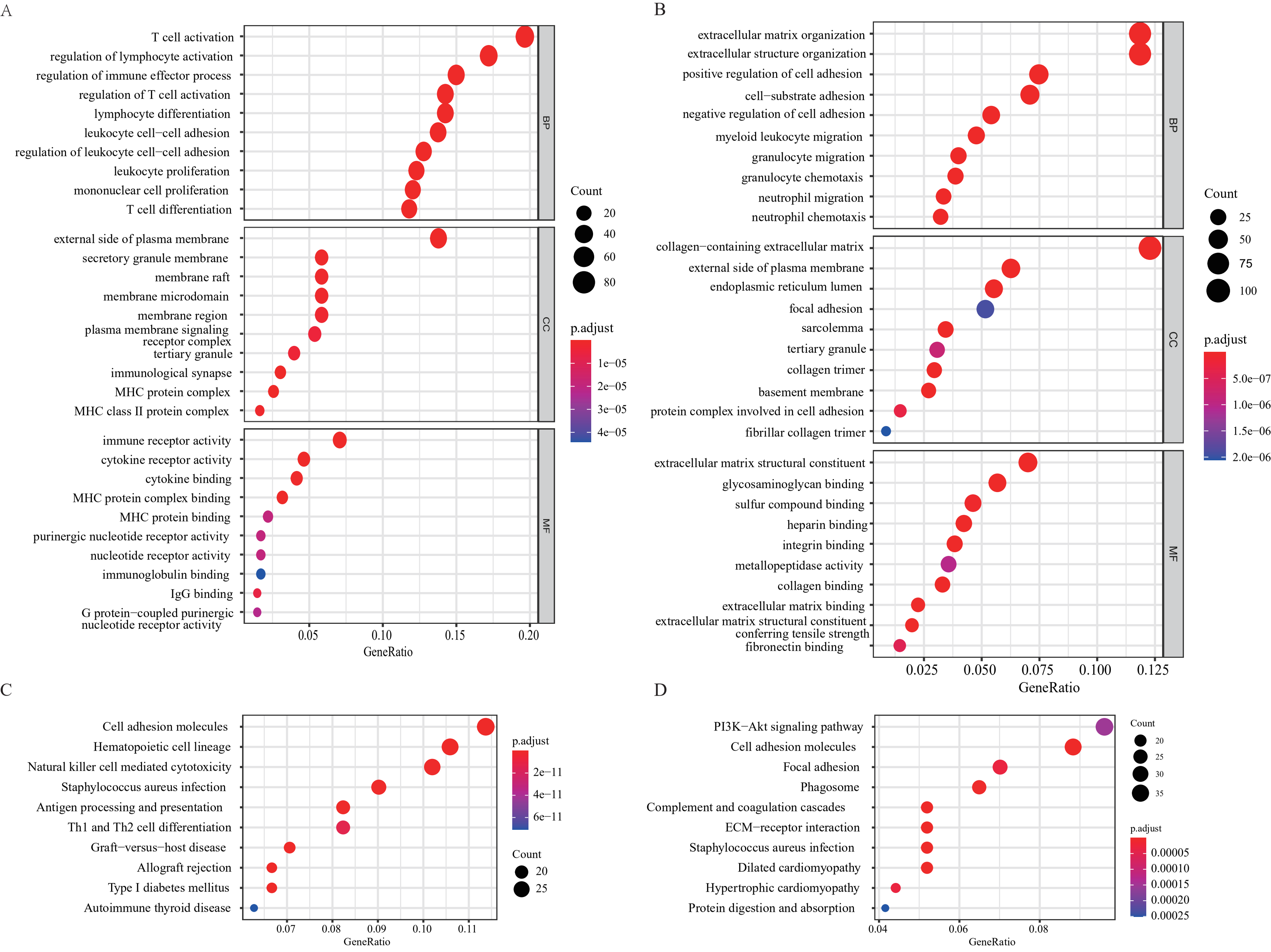

Supplementary Figure S3. The function enrichment analysis results of DEGs. (A) GO analysis of DEGs based comparation of on high/low immune score; (B) GO analysis of DEGs based on comparation of high/low stromal score; (C) KEGG analysis of DEGs based on comparation of high/low immune score; (D) KEGG analysis of DEGs based on comparation of high/low stromal score.
