# Supplementary material for: Identification of the Prognostic Value of Tumor Microenvironment-Related Genes in Esophageal Squamous Cell Carcinoma
Source: Front Mol Biosci. 2020 Dec 14;7:599475. doi: 10.3389/fmolb.2020.599475 (PMC7767869; doi:10.3389/fmolb.2020.599475)
Supplement: Supplementary file 4 [file Data_Sheet_4.PDF]

A

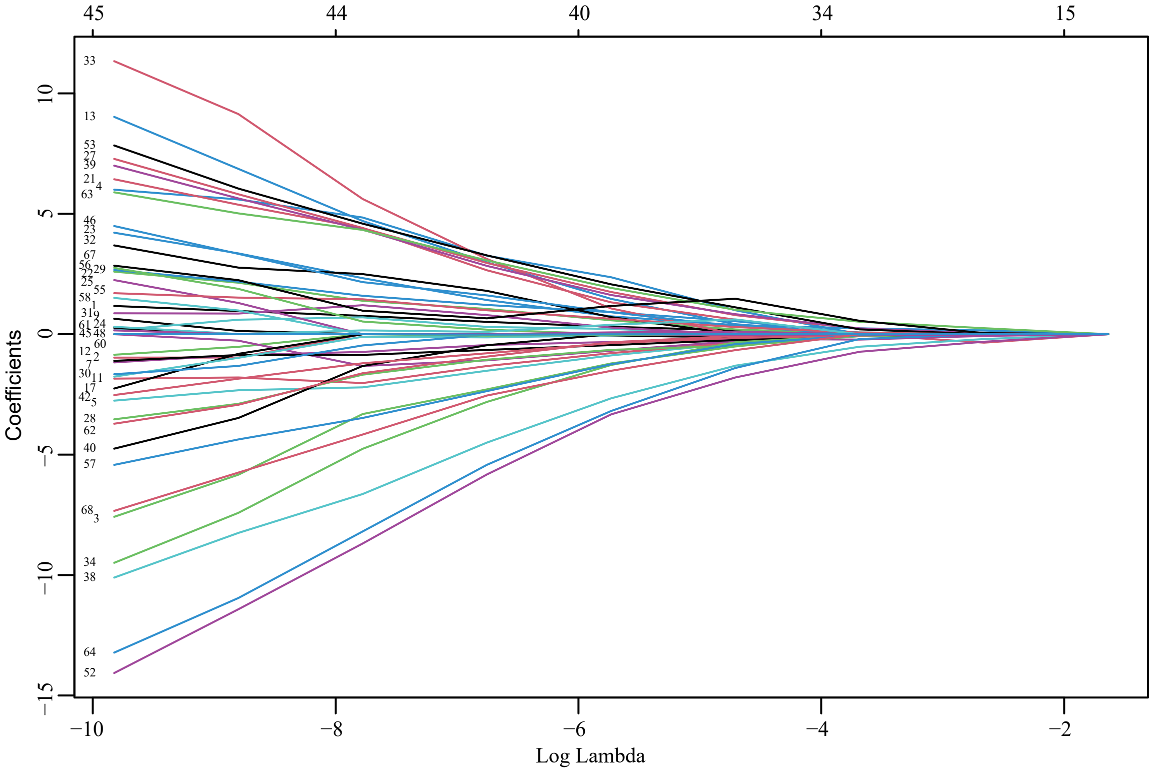

B

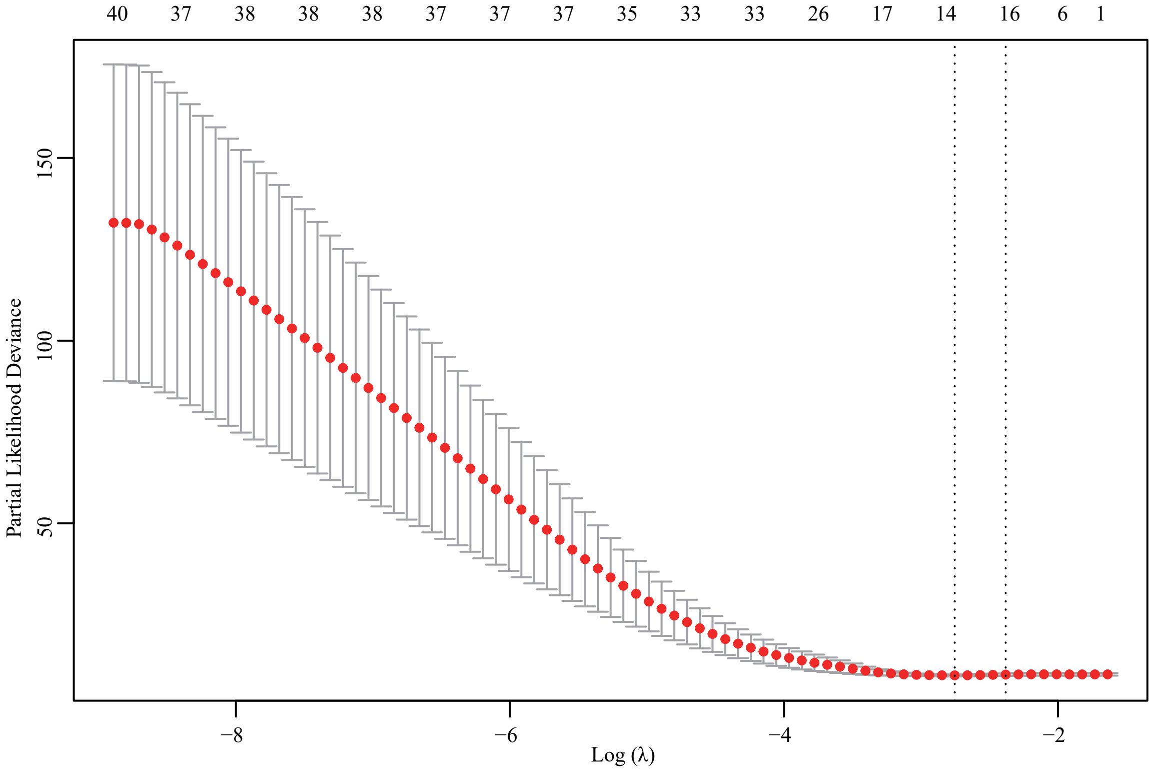

Supplementary Figure S4. Construction of the metabolic prognostic signature by LASSO regression. (A) Thee coefficient profiles of 67 prognostic genes. (B) The tuning parameter selection plot of LASSO regression. The dotted lines, respectively, represented the minimum and 1-SE lambda for the optimal volume of variables.
