# Supplementary material for: Identification of the Prognostic Value of Tumor Microenvironment-Related Genes in Esophageal Squamous Cell Carcinoma
Source: Front Mol Biosci. 2020 Dec 14;7:599475. doi: 10.3389/fmolb.2020.599475 (PMC7767869; doi:10.3389/fmolb.2020.599475)
Supplement: Supplementary file 5 [file Data_Sheet_5.PDF]

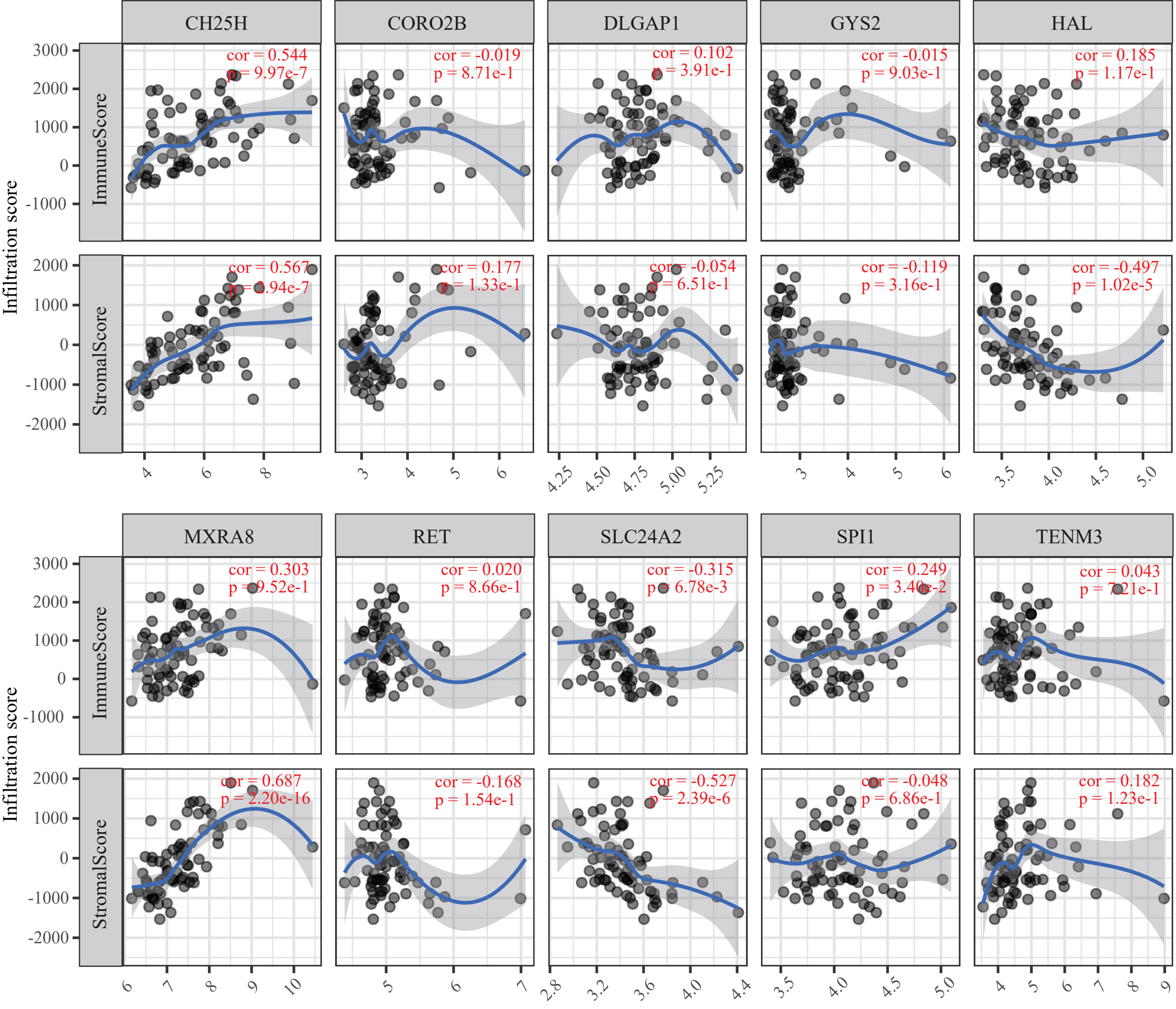

Supplementary Figure S5. The correlation between the expression of partial microenvironment related prognostic genes and immune/stromal scores
