# Supplementary material for: Identification of the Prognostic Value of Tumor Microenvironment-Related Genes in Esophageal Squamous Cell Carcinoma
Source: Front Mol Biosci. 2020 Dec 14;7:599475. doi: 10.3389/fmolb.2020.599475 (PMC7767869; doi:10.3389/fmolb.2020.599475)
Supplement: Supplementary file 6 [file Table_1.DOCX]

SUPPLEMENTARY TABLES

Table S1 67 genes who have tight connection with overall survival

| Gene Name | Logrank p value | Up/Down regulation |
| --- | --- | --- |
| FLJ21551 | 0.048375541 | up |
| LOC654142 | 0.010652747 | up |
| ADAMTS16 | 0.046551627 | up |
| AEBP1 | 0.00456595 | up |
| APBB1IP | 0.03705274 | up |
| ASB2 | 0.048103805 | up |
| AXL | 0.00331131 | up |
| MILR1 | 0.017695114 | up |
| LDLRAD4 | 0.006790831 | up |
| LOC51089 | 0.024872019 | up |
| CCL18 | 0.013228179 | up |
| CD14 | 0.029431149 | up |
| CD4 | 0.00767319 | up |
| CD86 | 0.000659209 | up |
| CDH11 | 0.004819015 | up |
| CH25H | 0.013854384 | up |
| CORO2B | 0.007962437 | up |
| CSF1R | 0.010459705 | up |
| CTSL | 0.014796095 | up |
| DYSF | 0.000362542 | up |
| ENPP2 | 0.006505049 | up |
| ENPP4 | 0.023048385 | up |
| FAM124A | 0.019519377 | up |
| FBP1 | 0.012791003 | up |
| FCER1G | 0.004950471 | up |
| FGL2 | 0.018761542 | up |
| GADL1 | 0.011443379 | up |
| GFRA2 | 0.004598378 | up |
| GJA5 | 0.023727549 | up |
| GPC6 | 0.005291962 | up |
| HLA-DRA | 0.04988788 | up |
| KCND2 | 0.027400064 | up |
| LAIR1 | 0.028936782 | up |
| LILRA6 | 0.019615658 | up |
| MEIS3 | 0.047215671 | up |
| MFAP5 | 0.014045744 | up |
| MMP16 | 0.048132997 | up |
| MS4A4 | 0.013123169 | up |
| LOC64166 | 0.00324815 | up |
| MS4A7 | 0.015084165 | up |
| MXRA8 | 0.033175933 | up |
| MYADM | 0.048412767 | up |
| NCKAP1L | 0.003976393 | up |
| NPTX1 | 0.043509359 | up |
| TENM3 | 0.031733901 | up |
| PDE1A | 0.010159046 | up |
| PIK3AP1 | 0.04520987 | up |
| LOC112491 | 0.00805645 | up |
| PLP1 | 0.027403441 | up |
| RELN | 0.0258398 | up |
| RET | 0.036709149 | up |
| RGS1 | 0.025690349 | up |
| RNASE6 | 0.003208699 | up |
| SLC24A2 | 0.029021876 | up |
| SPI1 | 0.043142074 | up |
| SRGN | 0.033446036 | up |
| STAB1 | 0.040477693 | up |
| SYT11 | 0.037947017 | up |
| TIMP2 | 0.028217648 | up |
| AMN | 0.009294204 | down |
| CLDN17 | 0.030095592 | down |
| DLGAP1 | 0.036187876 | down |
| GYS2 | 0.043302969 | down |
| HAL | 0.045518023 | down |
| KLK14 | 0.048892467 | down |
| MEX3A | 0.01948304 | down |
| OTX1 | 0.019075943 | down |

Logrank p value, p value of logrank test; Up/Down regulation, up or down regulation for immune/stromal high score group when compared with low score group.

Table S2 The correlation between 13 key prognostic genes and infiltration of immune cells

| Immune Cell | ADAMTS16 | CH25H | CORO2B | DLGAP1 | GYS2 | HAL | LOC51089 | MXRA8 | NPTX1 | OTX1 | RET | SLC24A2 | SPI1 |
| --- | --- | --- | --- | --- | --- | --- | --- | --- | --- | --- | --- | --- | --- |
| B.cells.memory | -0.04 | **0.2** | 0.18 | -0.01 | 0.05 | -0.01 | **0.27** | -0.08 | 0.15 | **-0.48** | -0.04 | -0.09 | **0.36** |
| B.cells.naive | 0.04 | 0.04 | 0.05 | 0.15 | **0.24** | 0.06 | **-0.22** | -0.13 | -0.07 | 0.07 | 0.17 | -0.05 | -0.12 |
| Dendritic.cells.activated | -0.08 | -0.13 | -0.13 | -0.01 | -0.03 | -0.07 | **-0.25** | **-0.21** | **-0.22** | 0.07 | -0.1 | -0.07 | **-0.26** |
| Dendritic.cells.resting | 0.11 | -0.03 | 0.11 | -0.02 | 0.05 | -0.07 | -0.08 | 0.05 | 0.13 | -0.03 | 0.08 | 0.01 | -0.04 |
| Eosinophils | -0.17 | -0.08 | 0 | 0.11 | 0.11 | 0.13 | **-0.31** | -0.17 | -0.02 | 0.12 | -0.07 | -0.07 | **-0.33** |
| Macrophages.M0 | 0.13 | -0.05 | 0.06 | **-0.17** | -0.22 | -0.06 | -0.1 | **0.32** | 0.02 | -0.06 | 0.17 | **0.41** | 0 |
| Macrophages.M1 | 0.09 | 0.09 | -0.16 | 0.06 | 0.12 | -0.01 | **0.35** | 0.03 | 0.12 | -0.08 | 0.18 | 0.03 | **0.26** |
| Macrophages.M2 | 0.18 | **0.2** | **0.22** | -0.04 | -0.15 | 0 | **0.53** | 0.2 | **0.3** | -0.15 | 0.16 | 0.06 | **0.41** |
| Mast.cells.activated | **-0.38** | **-0.26** | -0.18 | -0.18 | -0.16 | -0.02 | **-0.3** | -0.2 | **-0.25** | 0.11 | **-0.21** | -0.14 | **-0.29** |
| Mast.cells.resting | 0.15 | **0.31** | **0.23** | **0.13** | 0.3 | 0.13 | 0.04 | -0.02 | 0.14 | 0.01 | 0.1 | **-0.22** | -0.05 |
| Monocytes | -0.07 | -0.02 | -0.11 | **0.31** | 0.16 | 0.08 | **-0.22** | **-0.29** | -0.2 | **0.26** | -0.2 | **-0.35** | **-0.24** |
| Neutrophils | **-0.25** | -0.18 | -0.19 | -0.16 | 0.1 | **0.22** | -0.17 | **-0.25** | 0 | -0.05 | -0.1 | -0.18 | -0.17 |
| NK.cells.activated | -0.12 | -0.11 | -0.11 | 0.09 | 0.02 | 0.04 | 0.01 | -0.15 | -0.08 | 0.09 | -0.09 | -0.08 | 0.05 |
| NK.cells.resting | -0.02 | 0.04 | -0.11 | 0.08 | -0.05 | -0.12 | -0.11 | -0.06 | -0.09 | 0.06 | -0.17 | -0.17 | -0.1 |
| Plasma.cells | 0.01 | -0.04 | -0.03 | 0.06 | 0.07 | 0 | -0.05 | -0.04 | -0.03 | -0.06 | -0.06 | 0 | -0.05 |
| T.cells.CD4.memory.activated | 0.1 | **0.3** | 0.03 | -0.06 | -0.06 | -0.16 | **0.29** | 0.11 | **0.1** | **-0.06** | 0 | 0.01 | **0.27** |
| T.cells.CD4.memory.resting | 0.14 | -0.17 | 0.05 | 0.07 | -0.03 | -0.17 | **-0.31** | 0.07 | -0.07 | 0.24 | 0 | 0.08 | **-0.31** |
| T.cells.CD4.naive | -0.03 | 0.01 | 0.02 | 0.05 | -0.06 | -0.18 | -0.15 | 0.05 | -0.26 | 0.07 | -0.17 | 0.07 | -0.14 |
| T.cells.CD8 | 0.01 | **0.23** | -0.01 | 0.01 | 0.07 | -0.04 | **0.46** | -0.02 | 0.19 | **-0.24** | -0.08 | -0.09 | **0.4** |
| T.cells.follicular.helper | -0.16 | -0.11 | **-0.23** | **0.23** | **0.35** | **0.33** | **-0.22** | **-0.3** | -0.16 | **0.22** | **-0.28** | **-0.27** | **-0.2** |
| T.cells.regulatory.Tregs. | -0.08 | **0.23** | 0.09 | 0.04 | 0.15 | 0.17 | **0.41** | -0.06 | 0.06 | **-0.34** | -0.1 | -0.14 | **0.45** |

The correlation between genes and infiltration of immune cells were tested by Pearson’s correlation analysis; the Pearson's correlation coefficients were showed in the table, and all P values less than 0.05 were displayed with bold.
